# Severe Chronic Constipation and Fecaloma in a Patient With Partial Agenesis of the Corpus Callosum: A Case Report

**DOI:** 10.7759/cureus.118495

**Published:** 2026-10-24

**Authors:** Ikenna Joel Edward, Pamela Momah, Nima Maharjan

**Affiliations:** 1 Medicine, University Hospitals of Morecambe Bay NHS Foundation Trust, Kendal, GBR; 2 Orthopaedics, University of Chester, Chester, GBR; 3 Trauma and Orthopaedics, Furness General Hospital, Barrow-in-Furness, GBR

**Keywords:** chronic constipation, corpus callosum (cc) agenesia, fecaloma, functional constipation, neurogenic constipation, partial agenesis of the corpus callosum

## Abstract

Symptoms of functional constipation are associated with dysregulation of the brain-gut axis (BGA). BGA stands as a framework that accounts for the bidirectional interaction between the central nervous system (CNS), autonomic nervous system (ANS), and the enteric nervous system (ENS). Considering these interactions, the brain (CNS) becomes central to bowel movement/defecation. Consequently, it becomes evident why conditions of the CNS or impacts on the CNS could lead to functional constipation.

This case report highlights the intricate relationship between the CNS and GI motility via the BGA. The clinical presentation of partial agenesis of the corpus callosum and refractory fecaloma demonstrates an association between structural neurological deficits and impaired visceral sensory processing, though further studies/investigation are needed to establish a causal link (direct) to the patient’s disrupted ability or physiological urge to defecate.

Acute management, such as manual evacuation, can be necessary in severe chronic constipation with fecaloma to prevent life-threatening stercoral perforation. Additionally, long-term management of neurogenic constipation should evolve beyond a standard approach. Individualized management strategies could be considered, which may involve transitioning from an urge-based to a time-based defecation routine, alongside adjuncts like prokinetic agents (e.g., prucalopride) and regular abdominal massage

## Introduction

Chronic constipation is a clinical syndrome or condition that is characterized by incomplete defecation, difficulty, or infrequent opening of the bowel [1,2]. Constipation also includes hard stools, straining, inability to pass stools, and fewer than three bowel movements a week [1,2]. 

In the UK, about 12.8 per 1000 people have been diagnosed by their general physician to have chronic constipation [3,4], thus contributing to a prevalence ranging from 10.1% to 15.3% [5]. This prevalence varies depending on the individual’s institutional settings or status. For example, individuals living in their own homes have a lower prevalence of constipation compared to those on long stays on the hospital wards (30%-40% and 70%, respectively). Additionally, women have about two to three times higher incidence compared to men, while the elderly and pregnant women are more at risk of constipation compared to the general population [3]. 

The impact of chronic constipation goes beyond physical symptoms, as recent studies have shown its impact on work productivity and quality of life [1,6]. Individuals suffering from chronic constipation tend to have poorer quality of life, thereby causing low work productivity [1,6]. This impact on quality of life is independent of laxative use/dependence [6]. 

Based on recent classifications, there are varied causes of chronic constipation, which could be grouped into structural, secondary (medication, medical conditions), and dysregulation of the brain-gut axis (BGA) [1,7,8]. Additionally, chronic constipation that is not attributed to secondary causes is classified under functional constipation. 

A meaningful percentage of studies published have associated the symptoms of functional constipation with dysregulation of the BGA [9]. BGA stands as a framework that accounts for the bidirectional interaction between the central nervous system (CNS), autonomic nervous system (ANS), and the enteric nervous system (ENS) [9]. In the BGA, the CNS regulates the ENS through the ANS, while the ENS has a direct effect on the gut through local inhibitory or excitatory neurons [9]. To explain these interactions, defecation will be used as an example.

In the rectum, local stretch receptors are activated when the rectum is distended or filled with stool, liquid, or gas [10]. This rectal distension generates afferent sensory signals that are relayed to the CNS, contributing to the conscious urge to defecate [10]. Concurrently, the distension triggers the rectoanal inhibitory reflex, which mediates the involuntary relaxation of the internal anal sphincter [10]. Lastly, conscious neurological oversight dictates the final step and determines the appropriate timing for bowel evacuation; the brain coordinates the voluntary relaxation of the pelvic floor and external anal sphincter muscles rather than depending on a direct parasympathetic response [10]. 

Considering these interactions, the brain (CNS) is central to bowel movement/defecation. Consequently, it becomes evident why conditions of the CNS or impacts on the CNS could lead to functional constipation. In view of this, this case report will use a real case scenario of a patient with chronic constipation to highlight the association between dysregulation of the BGA and chronic constipation. 

## Case presentation

A 29-year-old female, a frequent attender to the emergency department (ED) for chronic constipation, presented to the ED with complaints of progressive generalized abdominal pain and a month-long history of difficulty in passing stool. She recounted her inability to feel the urge to defecate and only sought medical care when she developed nausea, severe cramping abdominal pain, vomiting, and early satiety. She reported low urine output with urgency and respiratory discomfort (could not take deep breaths) but denied any fever, hematochezia, or melena. In the past, she had attended the ED on multiple occasions for the same problem and was advised on lifestyle modifications and prescribed laxatives. She tried both lifestyle modifications and laxatives, including MiraLAX, Bisacodyl, Macrogol, and phosphate enemas, with no improvement in her symptoms and condition. 

Regarding her past medical and surgical history, she was known to have had partial agenesis of the corpus callosum since childhood with sensory processing differences and mild developmental coordination disorder. Additionally, she had a long history of chronic constipation previously diagnosed as functional constipation. The patient also reported being surgically treated for placental abruption three months prior. 

She was in severe discomfort during examination. Her vitals were stable except for mild tachycardia. Abdominal examination revealed a soft abdomen, but it was markedly distended with severe generalized tenderness and guarding, but no rigidity or peritonitis. A hard, non-mobile mass was palpated at the left iliac fossa extending to the suprapubic region. She could not tolerate a digital rectal examination because of pain. 

Based on the severity of her symptoms, full blood count, C-reactive protein (CRP), renal profile, liver profile, and computed tomography of the abdomen and pelvis (CTAP) with contrast were requested. Laboratory tests revealed an elevated CRP with normal white blood cell and renal function tests. Table 1 presents the patient's laboratory test trends throughout admission. 

**Table 1:** Routine blood reports

Test	Units	Reference Range	Day 2	Day 1
C-reactive protein	mg/L	0.0 - 5.0	31.3	117.7
Basophils	10*9/L	0.02 - 0.10	0	0
Eosinophil count	10*9/L	0.02 - 0.50	0.5	0.4
White blood cells	10*9/L	4 - 11	9.5	10.3
Neutrophils	10*9/L	2 - 7	5.4	6.7
Platelet count	10*9/L	150 - 400	330	238
Hemoglobin	g/dL	12.1-15.1	11.6	9.9
Alanine aminotransferase level	Int Unit/L	10 - 130	17	18
Albumin	g/L	35-50	34	29
Alkaline phosphatase	Int Unit/L	44 -147	107	85
Bilirubin	umol/L	< 21	14	23
Chloride	mmol/L	95-108	103	105
Creatinine	umol/L	60 - 110	65	62
Potassium	mmol/L	3.5-5.3	4.5	4.4
Sodium	mmol/L	133-146	137	139
Urea	mmol/L	2.5-7.8	2.9	2.5

Imaging findings

CTAP with contrast showed a fecal mass causing a mass effect on the bladder. No evidence of perforation or bowel strangulation was noted. Consistently, a diagnosis of fecaloma (fecal impaction) was made (Figures 1, 2).

**Figure 1:**
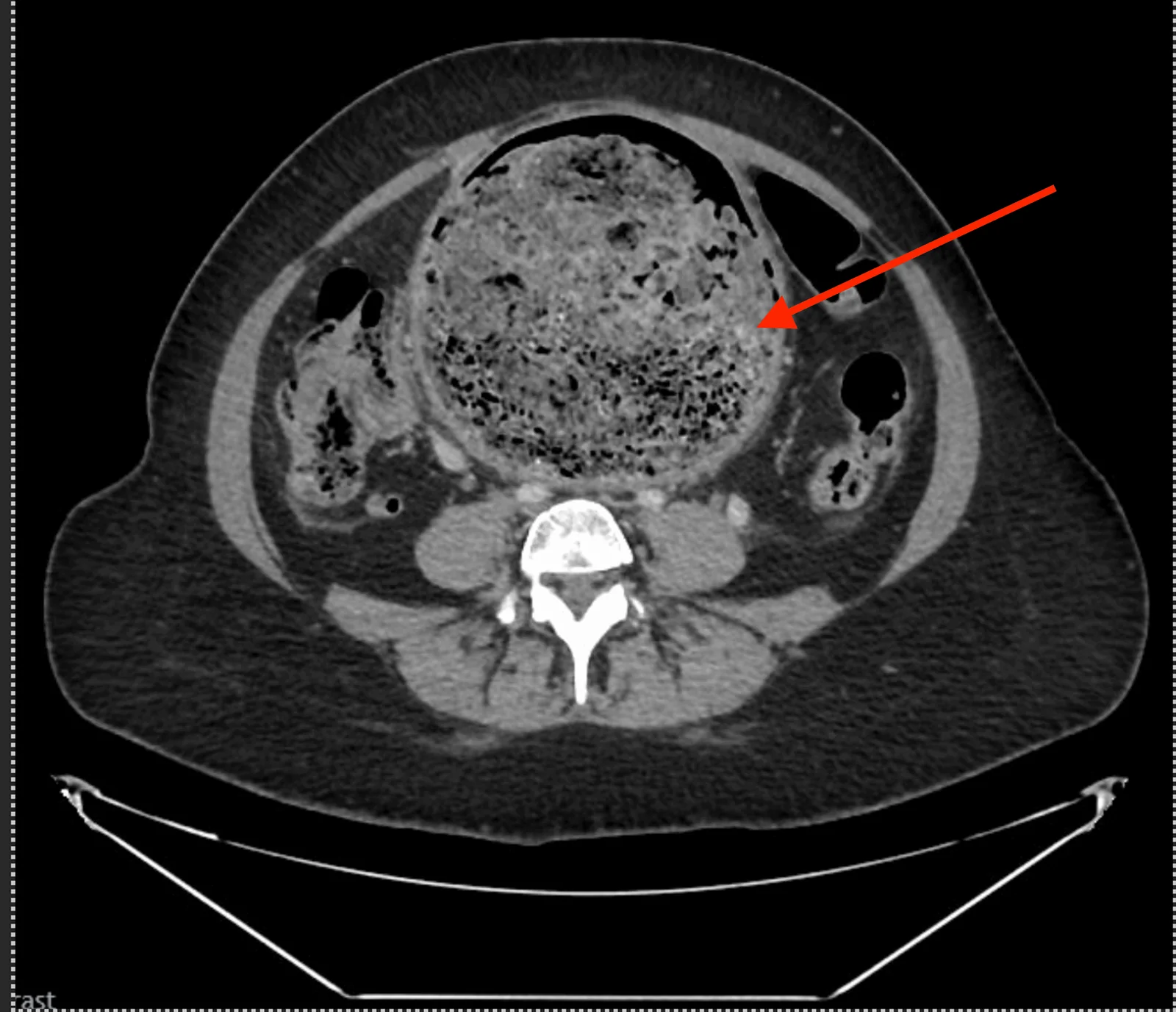
CT of the abdomen and pelvis with contrast; the red arrow shows a fecal mass occupying the rectum, extending proximally.

**Figure 2:**
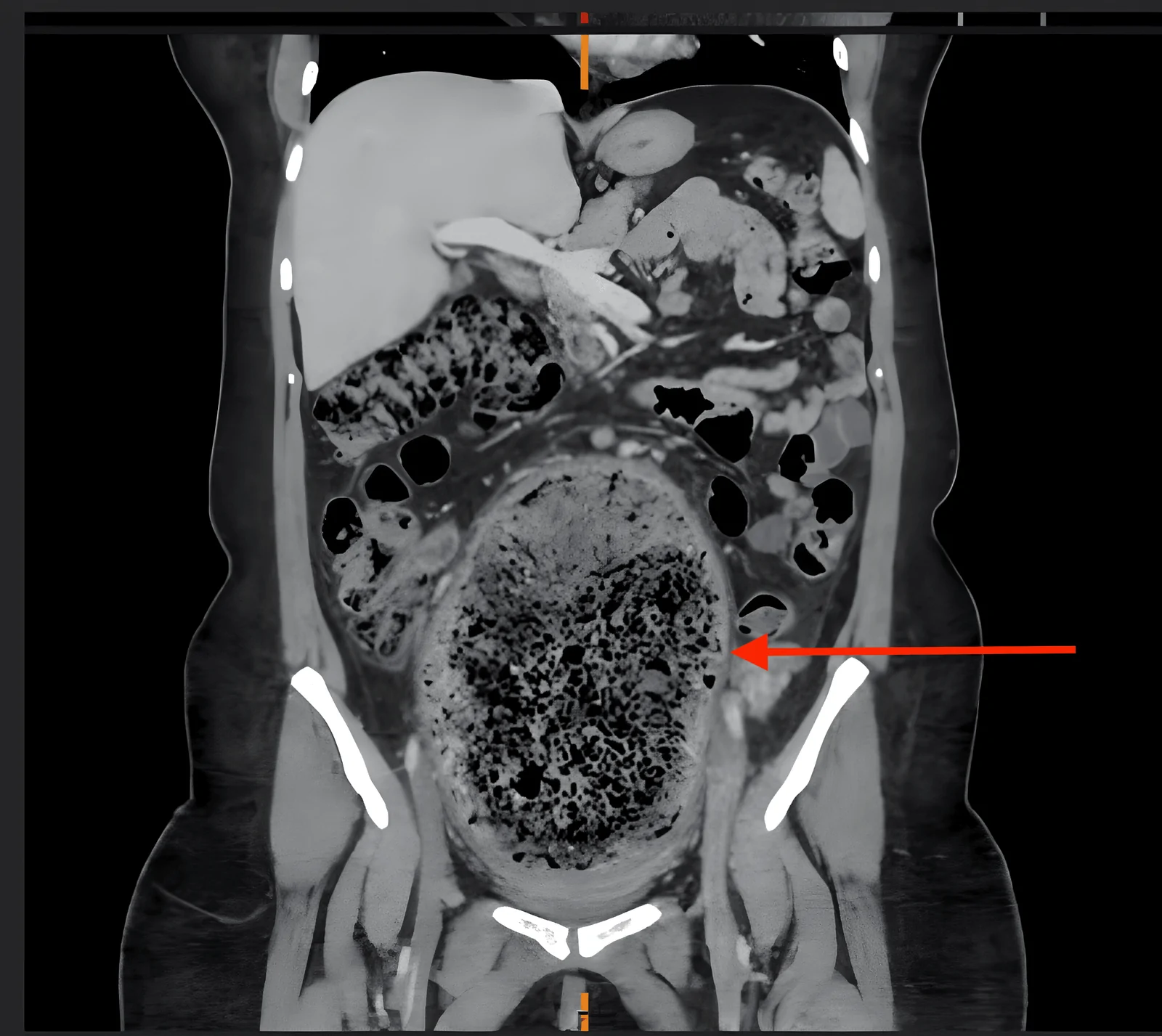
CT image The mass displays a mottled, heterogeneous appearance with a mixture of soft tissue density and trapped gas pockets (the classic "fecal impaction" or "colonic content" signature), characteristic of hard, impacted stool. The red arrow points to the lateral border of this large fecal mass, which exerts substantial extrinsic pressure on surrounding pelvic and lower abdominal structures, notably compressing the urinary bladder located inferiorly (this could be attributed to her urinary symptoms).

Management and treatment 

Given the definitive CT findings, worsening clinical symptoms, and failure of conservative laxative therapy, she was taken to the operating theater for manual evacuation of the fecaloma under general anesthesia. As complete clearance of the impacted fecal material could not be achieved intraoperatively, a large-volume phosphate enema regimen was subsequently administered postoperatively to facilitate further evacuation. 

To evaluate the efficacy of these interventions and rule out acute complications, a postoperative abdominal X-ray was performed (Figure 3). Comparative analysis with the preoperative CT scan demonstrated a substantial reduction in fecal burden, with remaining material successfully restricted to the rectum and a resolution of the previous colonic distension. Furthermore, there was no evidence of free intraperitoneal gas (ruling out acute stercoral perforation), and the proximal colonic loops appeared non-dilated and decompressed.

**Figure 3:**
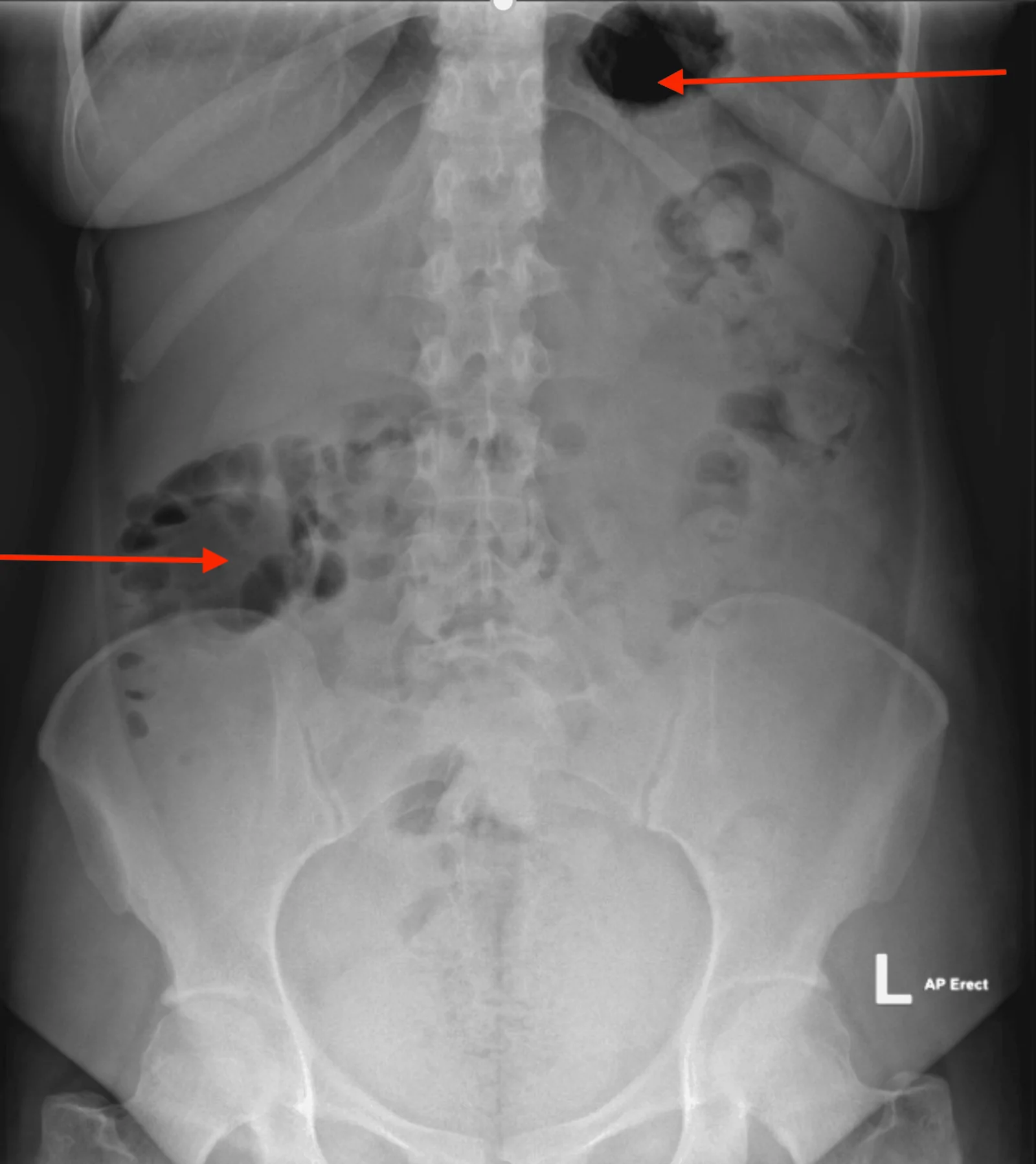
Postoperative abdominal X-ray. Compared to the preoperative CT, there is an absence of fecaloma, which was previously distending the lower abdomen and pelvis, confirming the successful manual evacuation. Left upper arrow: normal bowel pattern; Right lower arrow: loops of the large bowel containing normal gaseous distribution, also demonstrating decompression.

Outcome and discharge

Postoperatively, the patient's acute symptoms, including respiratory discomfort and cramping abdominal pain, were relieved. She developed expected minor postoperative complications, including transient hypotension, anal orifice pain, and mild bruising, which were successfully managed with fluid resuscitation, withholding blood pressure medications, and topical hydrocortisone/zinc oxide (Anusol). 

Despite the resolution of the acute impaction, clinical observation inferred that the patient continued to experience absent or severely delayed spontaneous bowel movements. It must be noted that this was a clinical inference based on ward observation; no formal colonic transit or motility studies were performed during this admission to objectively quantify colonic inertia. 

Upon discharge, an individualized treatment regimen was planned. She was recommended to transition to a time-based defecation routine (attempting to open her bowels one hour after meals) rather than relying on a physiological urge. The prokinetic agent prucalopride was prescribed to stimulate colonic transit, and regular abdominal massage was recommended. An outpatient gastroenterology referral was placed. Crucially, post-discharge follow-up data are unavailable, and the patient is awaiting a specialist neurogastroenterology assessment for further evaluation. Therefore, the long-term therapeutic efficacy of this planned discharge regimen is entirely unknown. 

## Discussion

Chronic constipation affects individuals of all ages, genders, and cultural backgrounds [6]. While its complications can occasionally be life-threatening, its daily impact on patient quality of life and work productivity is profoundly debilitating [11]. 

The etiology of chronic constipation is complex and multifactorial. In functional cases, symptoms are closely linked to a dysregulation of the BGA [9,12]. The BGA is the central neuro-enteric framework that ensures efficient gastrointestinal motility. Thus, disruptions originating from the brain, spinal cord, or the intrinsic colonic nervous system can result in the same clinical endpoint, severe constipation [9]. However, before attributing treatment-resistant constipation to a disorder of gut-brain interaction, it is imperative to exhaust all differential diagnoses. Other causes of chronic constipation, including primary structural bowel problems, endocrine disorders (such as hypothyroidism), metabolic disturbances, and medication-induced dysmotility, must be carefully distinguished and ruled out [2]. 

In this case, the patient presented with a history of partial agenesis of the corpus callosum. Neuroimaging and clinical evidence suggest an association between corpus callosum abnormalities and functional gastrointestinal disorders, including severe colonic dysmotility and irritable bowel syndrome with constipation [1,13]. The corpus callosum plays a vital role in integrating sensory information, processing emotions, and regulating cognitive and cortical functions [1]. Its dysfunction has been implicated in conditions characterized by altered pain and sensory perception, such as fibromyalgia and chronic pain [1]. Consequently, while partial agenesis of the corpus callosum is not currently established as a direct primary cause of chronic constipation, it is proposed here as a contributing factor. The structural deficit may impair the visceral sensory processing required to recognize rectal distension, thereby disrupting the physiological urge to defecate. However, alternative multifactorial etiologies for her refractory constipation cannot be definitively excluded. 

When chronic constipation goes untreated, it can lead to severe complications. It accounts for 50% of fecal impactions and 3.2% of colonic perforations [14,15]. Fecalomas increase intraluminal pressure, which can cause ischemia and colitis, ultimately progressing to stercoral perforation if left unmanaged [14,15]. The sigmoid and rectosigmoid colon are at the highest risk for this complication due to narrow diameters, firm stool consistency, and relatively poor vascularization [14,16]. As perforation is a surgical emergency, patients with chronic constipation and refractory fecalomas require urgent physical examination and appropriate imaging [14-16]. 

The management of neurogenic and treatment-resistant constipation typically begins with conservative optimization of bowel regimens and progresses to more invasive approaches [17]. Conservative steps include high-fiber diets, medication reviews, and the establishment of strict bowel care routines [17]. When these fail, adjuncts such as abdominal massage, laxatives, and manual evacuation may be necessary. For patients unresponsive to these measures, transanal irrigation or electrical stimulation can often prevent the need for invasive surgeries like colonic resection with or without stoma formation [17]. 

In this patient's case, acute management required manual evacuation under general anesthesia due to the failure of conservative therapies. Postoperatively, her care strictly adhered to protocols facilitating complete bowel clearance. Given the suspected neurogenic component of her condition, long-term management requires customized, individualized options rather than standard prescriptive protocols. She was counseled on establishing a structured bowel routine, specifically transitioning to time-based defecation, and was prescribed pharmacological adjuncts to stimulate colonic transit as part of a multidisciplinary approach.

A notable limitation of this case report is the absence of specialized neurogastroenterological testing to definitively confirm the causal pathway between the patient's corpus callosum agenesis and her colonic dysmotility. To objectively establish a central sensory processing deficit, future investigations could utilize cortical sensory evoked potentials or functional MRI paired with rectal balloon distension. These modalities can map real-time cortical responses to visceral stimulation, thereby visualizing disrupted afferent signaling. Additionally, high-resolution anorectal manometry with sensory threshold testing could be employed to formally quantify rectal hyposensitivity, further supporting BGA dysregulation as the primary etiology for her absent urge to defecate. Finally, the complete absence of post-discharge follow-up data prevents any assessment of the efficacy or benefit of the recommended long-term management strategies, including time-based defecation and prucalopride therapy. 

## Conclusions

This case suggests a possible association between partial agenesis of the corpus callosum and severe, refractory chronic constipation. While we hypothesize that structural CNS anomalies may impair the visceral sensory processing necessary for normal defecation, a single case cannot establish causality or definitively confirm this mechanism. The primary clinical takeaway is that treatment-resistant constipation complicated by massive fecaloma requires urgent mechanical intervention to avert life-threatening perforation, followed by the careful planning of highly individualized maintenance therapies. Although a tailored regimen incorporating prokinetics and time-based defecation was recommended upon discharge, the absence of longitudinal follow-up data leaves its therapeutic efficacy unknown. Ultimately, managing complex constipation warrants an exhaustive look into secondary neurological factors, but validating these intricate brain-gut interactions relies on future, dedicated pathophysiological research. 
